# Evaluation of Film-Forming Properties of α-1,3-Glucan Obtained from “Chicken of the Woods” Mushroom (*Laetiporus sulphureus*): Film Development, Characterization, and Biodegradation Assessment

**DOI:** 10.3390/molecules30071619

**Published:** 2025-04-04

**Authors:** Dariusz Kowalczyk, Barbara Gieroba, Katarzyna Niedźwiadek, Mikołaj Krysa, Anna Sroka-Bartnicka, Adam Waśko, Ewa Ozimek, Aleksandra Ściegienna, Monika Basiura-Cembala, Waldemar Kazimierczak, Adrian Wiater

**Affiliations:** 1 Department of Biochemistry and Food Chemistry, Faculty of Food Sciences and Biotechnology, University of Life Sciences in Lublin, Skromna 8, 20-704 Lublin, Poland; dariusz.kowalczyk@up.lublin.pl (D.K.); katarzyna.lupina@gmail.com (K.N.); 2 Independent Unit of Spectroscopy and Chemical Imaging, Medical University of Lublin, Chodźki 4a, 20-093 Lublin, Poland; barbaragieroba@umlub.pl (B.G.); mikolaj.krysa@umlub.pl (M.K.); anna.sroka@umlub.pl (A.S.-B.); 3 Department of Biotechnology, Microbiology and Human Nutrition, Faculty of Food Science and Biotechnology, University of Life Sciences in Lublin, Skromna 8, 20-704 Lublin, Poland; adam.wasko@up.lublin.pl; 4 Department of Industrial and Environmental Microbiology, Institute of Biological Sciences, Faculty of Biology and Biotechnology, Maria Curie-Skłodowska University, Akademicka 19, 20-033 Lublin, Poland; ewa.ozimek@mail.umcs.pl (E.O.); ola.sciegienna04@gmail.com (A.Ś.); 5 Institute of Engineering Sciences, Faculty of Materials, Civil and Environmental Engineering, University of Bielsko-Biala, Willowa 2, 43-309 Bielsko-Biała, Poland; mbasiura@ubb.edu.pl; 6 Department of Biomedicine and Environmental Research, Faculty of Medicine, John Paul II Catholic University of Lublin, Konstantynów 1J, 20-708 Lublin, Poland; waldemar.kazimierczak@kul.pl

**Keywords:** *Laetiporus sulphureus* mushroom, α-1,3-glucan, biodegradable film, molecular structure, tensile strength, water vapor permeability

## Abstract

Unlike many biopolymers, α-1,3-glucan (α-1,3-GLU) is water-insoluble, making it a promising candidate for the production of moisture-resistant films with applications in biodegradable packaging, biomedicine, and cosmetics. This study aimed to characterize the structural, physicochemical (water affinity, optical, mechanical), and biodegradation properties of a film made from α-1,3-GLU extracted from *Laetiporus sulphureus*. The film was fabricated through alkaline dissolution, casting, drying, washing to remove residual NaOH, and re-plasticization with a glycerol solution. FTIR and Raman spectroscopy confirmed the polysaccharide nature of the film, with predominant α-glycosidic linkages. The film exhibited a semi-crystalline structure and high opacity due to surface roughness resulting from polymer coagulation. Owing to re-plasticization, the film showed a high moisture content (~47%), high water solubility (81.95% after 24 h), and weak mechanical properties (tensile strength = 1.28 MPa, elongation at break ≈ 10%). Its water vapor permeability (53.69 g mm m^−2^ d^−1^ kPa^−1^) was comparable to other glycerol-plasticized polysaccharide films reported in the literature. The film supported the adhesion of soil microorganisms and target bacteria and was susceptible to degradation by *Trichoderma harzianum* and endo- and exo-α-1,3-glucanases, indicating its biodegradability. The limitations in its mechanical strength and excessive hydration indicate the need for improvements in the composition and methods of producing α-1,3-GLU films.

## 1. Introduction

The depletion of petroleum resources, along with the growing issues of plastic waste, microplastics, and harmful substances in synthetic materials, is driving the development and adoption of biopolymers from renewable sources for sustainable material production. Biopolymer-based films are emerging as eco-friendly alternatives to conventional plastics, with applications in packaging [1], agriculture (e.g., biodegradable mulches, seed coatings) [2], biomedicine (e.g., drug delivery, tissue engineering, wound healing) [3], and cosmetics (e.g., face masks, skin patches) [4]. However, a major challenge for biopolymers is their high sensitivity to moisture, which limits their mechanical strength, barrier properties, and overall durability. To address this, researchers are modifying biopolymers through blending with hydrophobic materials, cross-linking, and nanofiller incorporation [5,6] or exploring water-insoluble biopolymers.

In line with eco-design principles, the use of food resources for non-food purposes is being minimized due to the rising global food demand [7]. This has shifted the focus to unconventional biomass sources like inedible plants, algae, bacteria, and fungi [8]. Fungi, particularly mushrooms, are promising due to their rapid growth, adaptability, and low resource requirements [9,10]. They absorb CO_2_ during growth, reducing their carbon footprint, and are rich in bioactive compounds, making them valuable for the production of both materials and functional foods [11]. Fungal biopolymers include cell wall polysaccharides (e.g., cellulose, chitin), structural matrix biopolymers (e.g., α-glucans, β-glucans, glycoproteins), and heteropolysaccharides containing fucose, galactose, mannose, and xylose [12,13]. Among these, α-1,3-glucan (α-1,3-GLU), a water-insoluble polysaccharide, is abundant in *Laetiporus sulphureus* (chicken of the woods), an edible mushroom found on hardwood trees, particularly oaks, across North America, Europe, and Asia [14,15,16,17]. The mushroom forms large clusters of 5 to 50 caps, each reaching 10 to 40 cm or more in diameter in optimal conditions [14,16]. With α-1,3-GLU constituting up to 88% of its dry mass, *L. sulphureus* is a superior source of this biopolymer [17], which has potential applications in the manufacturinge of biodegradable films for packaging [18,19,20] and cosmetics (e.g., after conversion to α-1,3-GLU hydroxypropyltrimonium chloride) [21]. It is worth noting that other fungi typically have only 9–46% of α-1,3-GLU [15]. Films can be produced by dissolving α-1,3-GLU in solvents, e.g., a base (but also formic acid, aqueous tetraethylammonium hydroxide, or a mixture of dimethyl sulfoxide and LiCl), followed by casting or extrusion and tension drying. The base is removed by washing the film with a coagulation medium (e.g., methanol, water, or acids). Optionally, the resulting film can be plasticized by immersion in a solution of glycerol or ethylene glycol. α-1,3-GLU films are transparent or hazy, with adjustable mechanical properties and thermal stability up to 150 °C [18,19,20].

Despite their potential, there are no comprehensive studies on α-1,3-GLU films. This study aims to develop and characterize film made from α-1,3-GLU extracted from *L. sulphureus*, evaluating its structural, physicochemical (water affinity, optical, mechanical), and biodegradation properties to assess its advantages and limitations compared to other biopolymer films, with a particular focus on moisture sensitivity.

## 2. Results and Discussion

### 2.1. Microscopic Features of the Film-Forming Solution (FFS) and Film

**Figure 1:**
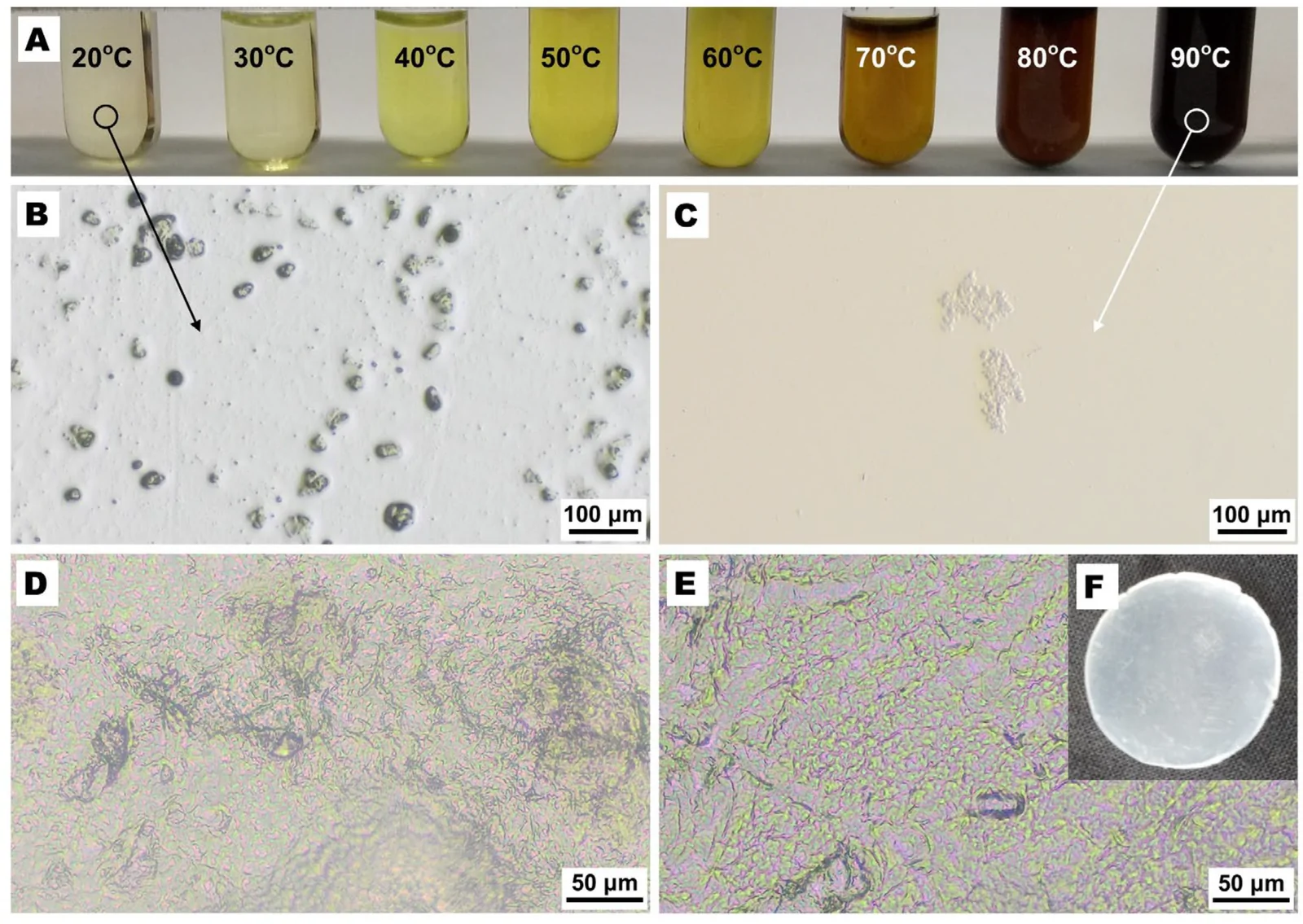
(**A**) Visual appearance of the film-forming solution (1M NaOH solution containing 5% α-1,3-glucan and 4% glycerol) after heating at various temperatures. Microscopic images of the film-forming solutions obtained at 20 °C (**B**) and 90 °C (**C**); microscopic images of the α-1,3-glucan film before (**D**) and after NaOH washing (**E**). Visual appearance of the α-1,3-glucan film after re-plasticization with an aqueous glycerol solution (**F**).

Despite the visual homogeneity of the FFS (Figure 1A), the microscopic observation revealed that, at 20 °C, α-1,3-GLU was not completely dissolved in 1M NaOH (Figure 1B). This finding was unexpected, as α-1,3-GLU is considered an alkali-soluble fraction of fungal cell walls [15]. It is possible that the limited dissolution was due to the high viscosity of the FFS, which hindered the migration of the solvent inside the larger and/or gel-layered α-1,3-GLU particles. A similar phenomenon has been observed in highly viscous solutions of methylcellulose [22,23] or chitosan lactate [24].

It is well known that many polysaccharides do not dissolve instantaneously, and external parameters such as agitation and heating above an upper critical solution temperature are required for complete dissolution [25,26]. Therefore, an additional experiment was conducted to determine the effect of heating (up to 90 °C) on the dissolution of α-1,3-GLU. As expected, the higher temperature resulted in better homogeneity of the FFS, and, finally, only sporadic gelled polymer remains were detected at 90 °C (Figure 1C). However, one unanticipated finding was that the heating induced rapid browning of the FFS (Figure 1A). This result may be explained by the fact that hot-alkali treatment decomposes linear glucans to glucose [27,28], which can then be rapidly caramelized [29]. In accordance with the present result, a previous study demonstrated that the color of amylose-rich corn starch extruded with increasing concentrations of an NaOH solution changed gradually from a translucent yellow to a dark brown [30]. It should be noted that 1M NaOH, as used in this study, has a pH of around 14. In conclusion, heating the FFS resulted in polymer degradation and the formation of an undesirable color, indicating that a non-thermal method or treatment (e.g., sonification) is necessary to achieve complete dissolution for effective film production using α-1,3-GLU.

NaOH, which enabled the dissolution of α-1,3-GLU, had to be removed from the film after its formation. Contrary to expectations, no NaOH crystals were found in the unwashed film (Figure 1D). Before (Figure 1D) and after NaOH washing (Figure 1E), the α-1,3-GLU film consisted of a layer of shapeless polymer crystal networks. Interestingly, in addition to predominant irregular polymer aggregates, some short fibrillar-like self-assemblies were observed in the film matrix.

**Figure 2:**
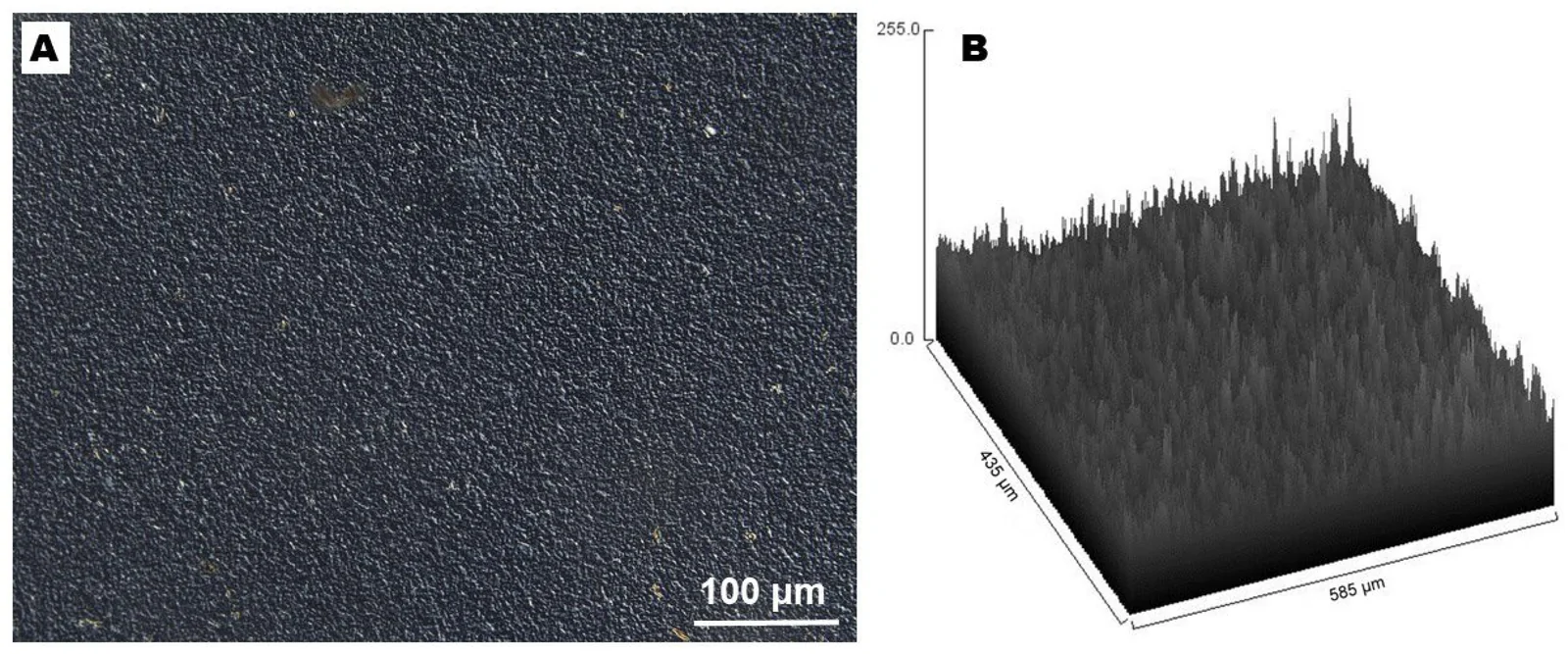
Differential interference contrast microscopy image (200× magnification) (**A**) and the corresponding 3D visualization (**B**) of the surface of the α-1,3-glucan film.

Differential interference contrast (DIC) microscopy enabled the visualization of the uniformly rough topography of the α-1,3-GLU film (Figure 2). The conversion of the 2D image to 3D allowed the calculation of the root-mean-square roughness (Rq), which was 26.24, indicating a significant level of roughness. For comparison, films with a very smooth topography, such as those containing casein, gelatin, gum Arabic, or pullulan, exhibit Rq values below 5 nm [24,31,32]. The Rq value observed in this study is comparable to those recorded for films containing corn or wheat starch, in which so-called “ghost starch granules” are present [24], and for films containing soybean hemicellulose—a polysaccharide characterized by low water solubility, leading to the formation of a granular microstructure on the surface [31]. The roughness of the α-1,3-GLU film reflects the disordered polymer crystal networks (Figure 1), which likely results from the coagulation of the polysaccharide upon contact with the water used for NaOH leaching and re-plasticization.

### 2.2. Structural Features of the Film

**Figure 3:**
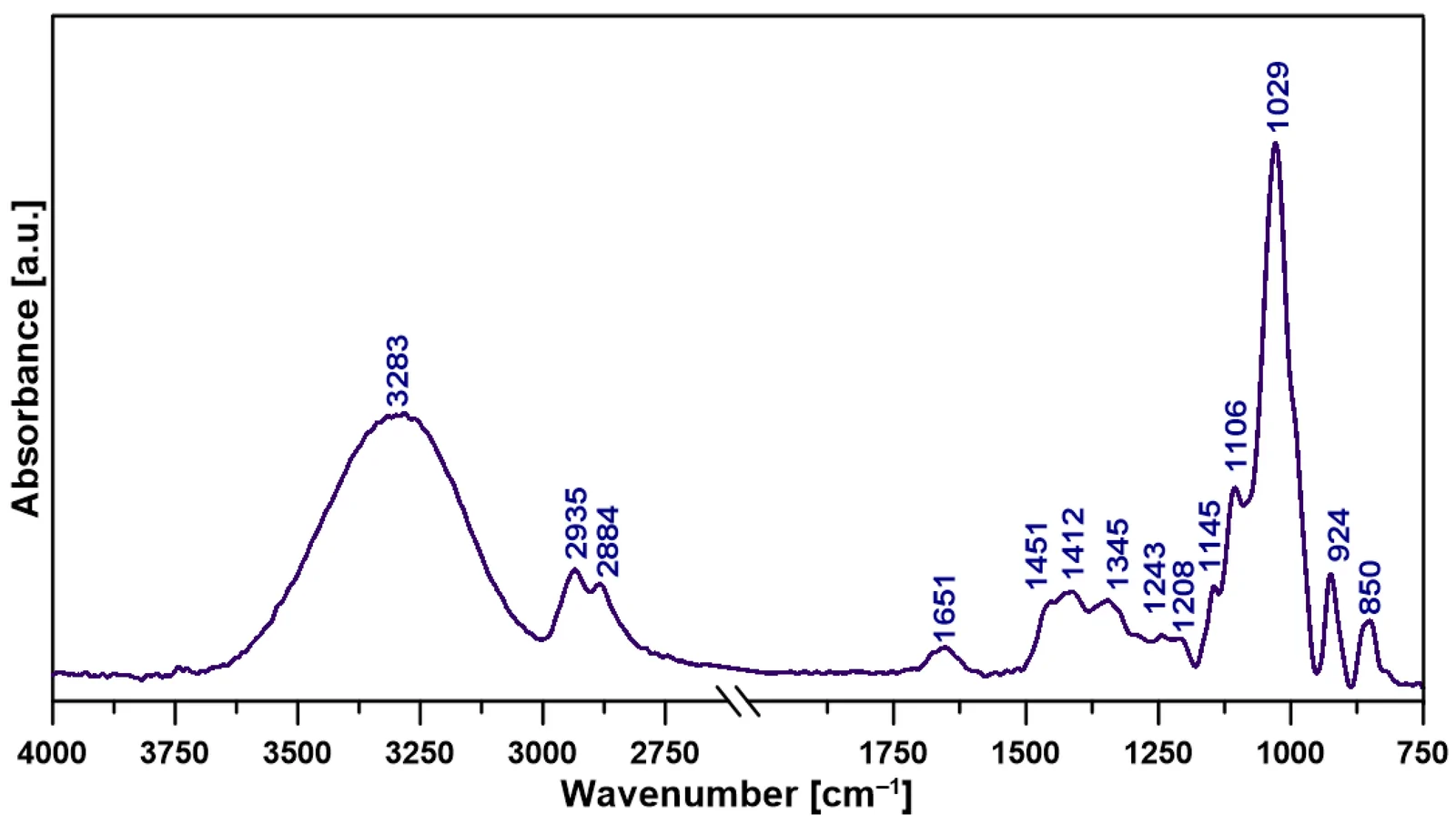
Attenuated total reflectance–Fourier transform infrared spectroscopy spectrum of the α-1,3-glucan film.

The attenuated total reflectance–Fourier transform infrared (ATR-FTIR) spectroscopy spectrum of the α-1,3-GLU film is presented in Figure 3, while the assignments of the characteristic bands are summarized in Table 1 [33,34,35]. The wide and intensive absorption peak located at 3283 cm^−1^ indicates -OH stretch of polysaccharide, glycerol, and water origin [36,37]. The bands at 1412 and 1345 cm^–1^ can be ascribed to vibrational modes of the -CH_3_, -CH_2_, and C-H groups [38], while the band at 1145 cm^−1^ is related to the C-O-C/C-C stretching modes of glycosidic linkage [33,35]. The band at 1067 cm^–1^ comes from the CH_2_OH stretch typical for polysaccharides [36], and the band at 1029 cm^–1^ is also in correlation with the presence of a glycosidic bond, i.e., C–O stretching vibrations [39]. Also, the 1200–1000 cm^−1^ spectral range corresponds to the C-O, C-C, and C-O-C stretching modes [38]. Finally, the band at 850 cm^−1^ is ascribed to out-of-plane bending vibrations of C-H in α-1,3-GLU units [33,34].

**Figure 4:**
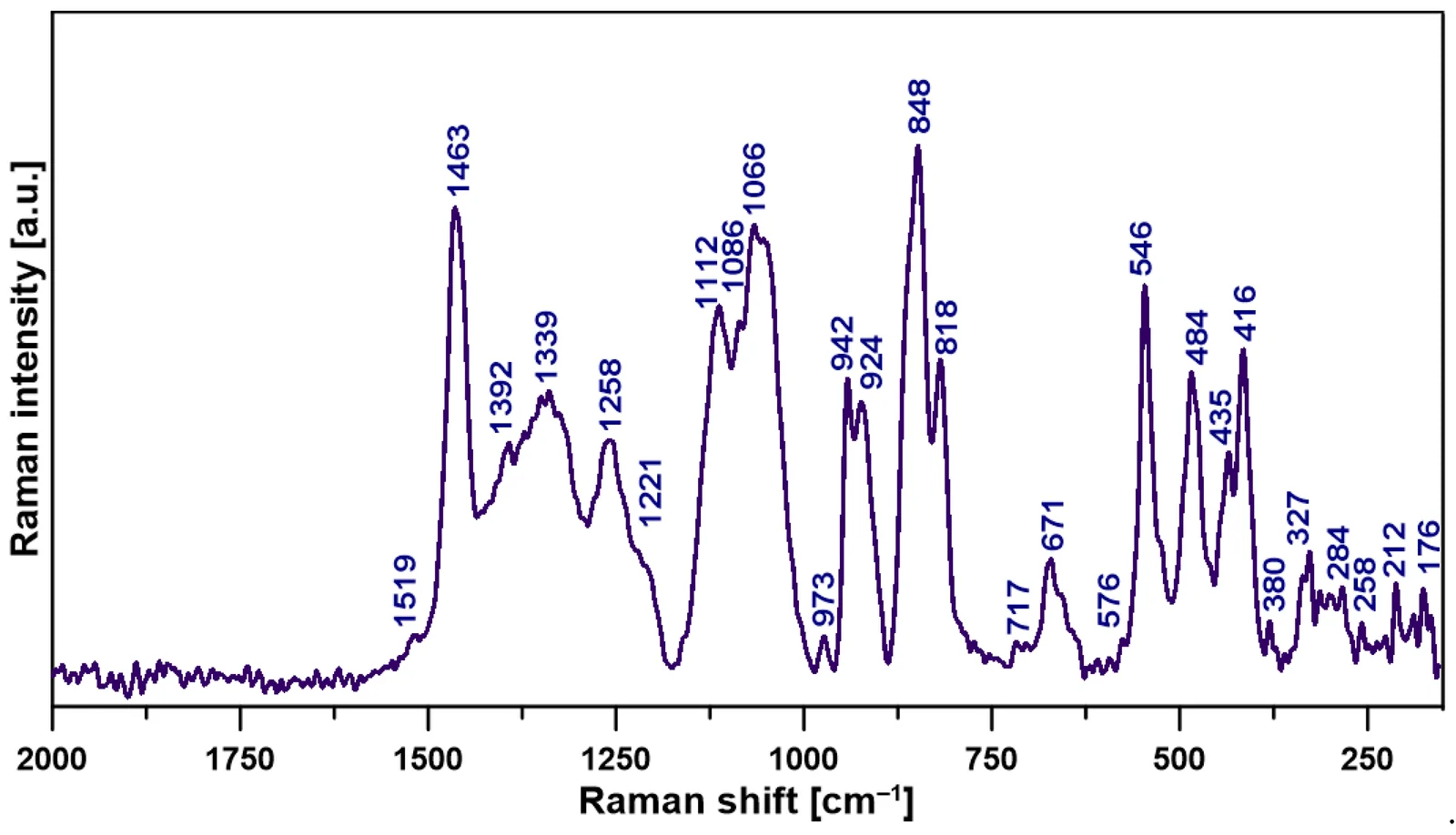
Raman spectrum of the α-1,3-glucan film.

The Raman spectrum of the α-1,3-GLU film with the corresponding band assignments is shown in Figure 4 and Table 2 [40,41,42]. The observed bands confirm the polysaccharide nature of the sample. Specifically, (i) the region between 1500 and 1200 cm^−1^ corresponds to COO− symmetric stretching, as well as the C-H deformational and bending modes; (ii) the spectral range between 1200 and 1000 cm^−1^ is attributed to the C-C stretching, C-O-C glycosidic linkage, and symmetric ring breathing modes; (iii) vibrations of side groups appear in the 1000–800 cm^−1^ range; (iv) the band below 460 cm^−1^ arises from C-C-C ring deformational modes; additionally, (v) the anomeric region (900–800 cm^−1^), typical for glycosidic bonds, is also present [41,43]. From the Raman spectra, it is possible to differentiate between α- and β-type linkages. Specifically, the C-H bending vibrations for the anomeric region between 905 and 885 cm^−1^ suggest β-type bonds, while those between 865 and 835 cm^−1^ indicate α-type bonds [44]. As shown in Figure 4, the bending associated with α-type bonds is more intense.

**Figure 5:**
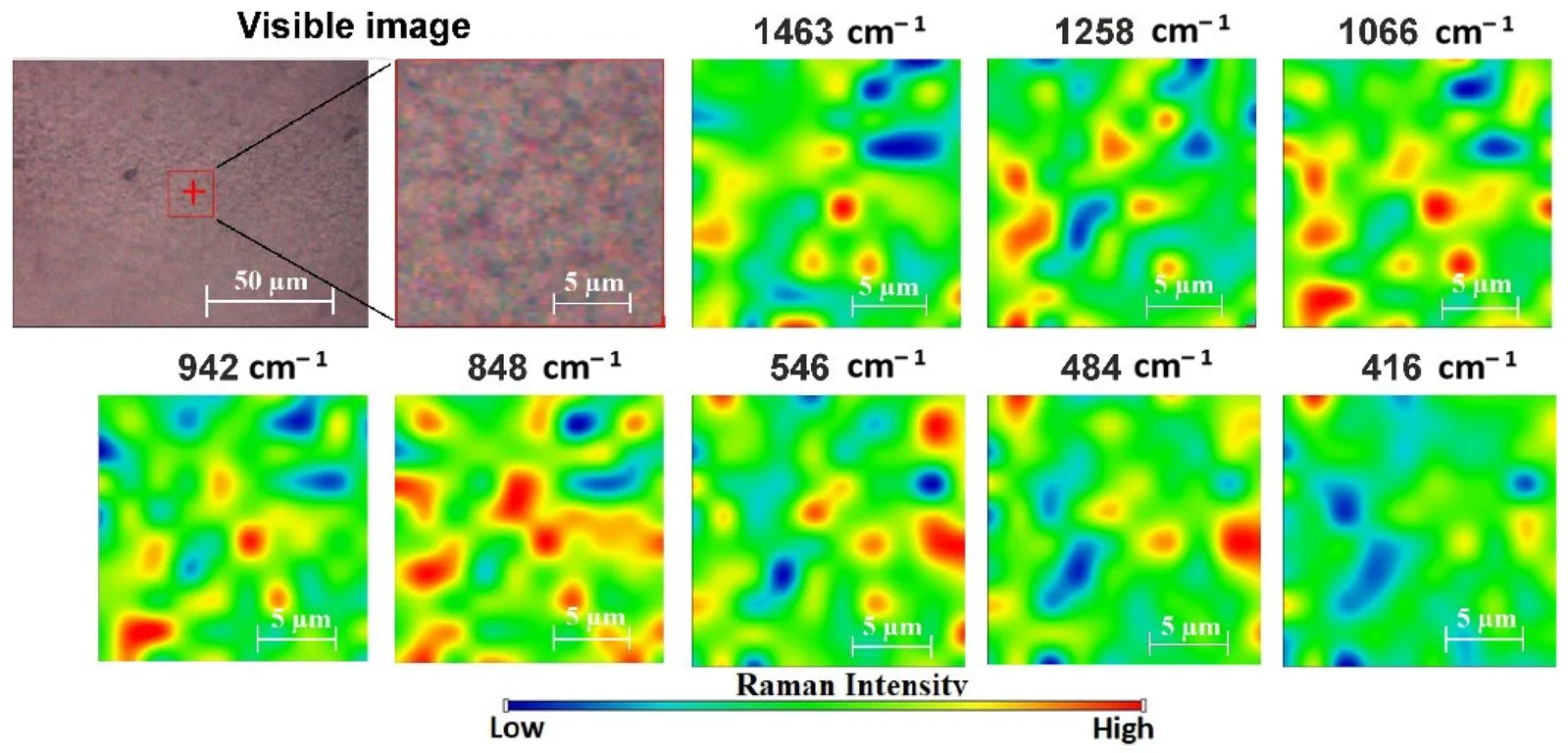
Raman microscopic visible images and chemical maps of compound distribution on the surface of the α-1,3-glucan film.

**Figure 6:**
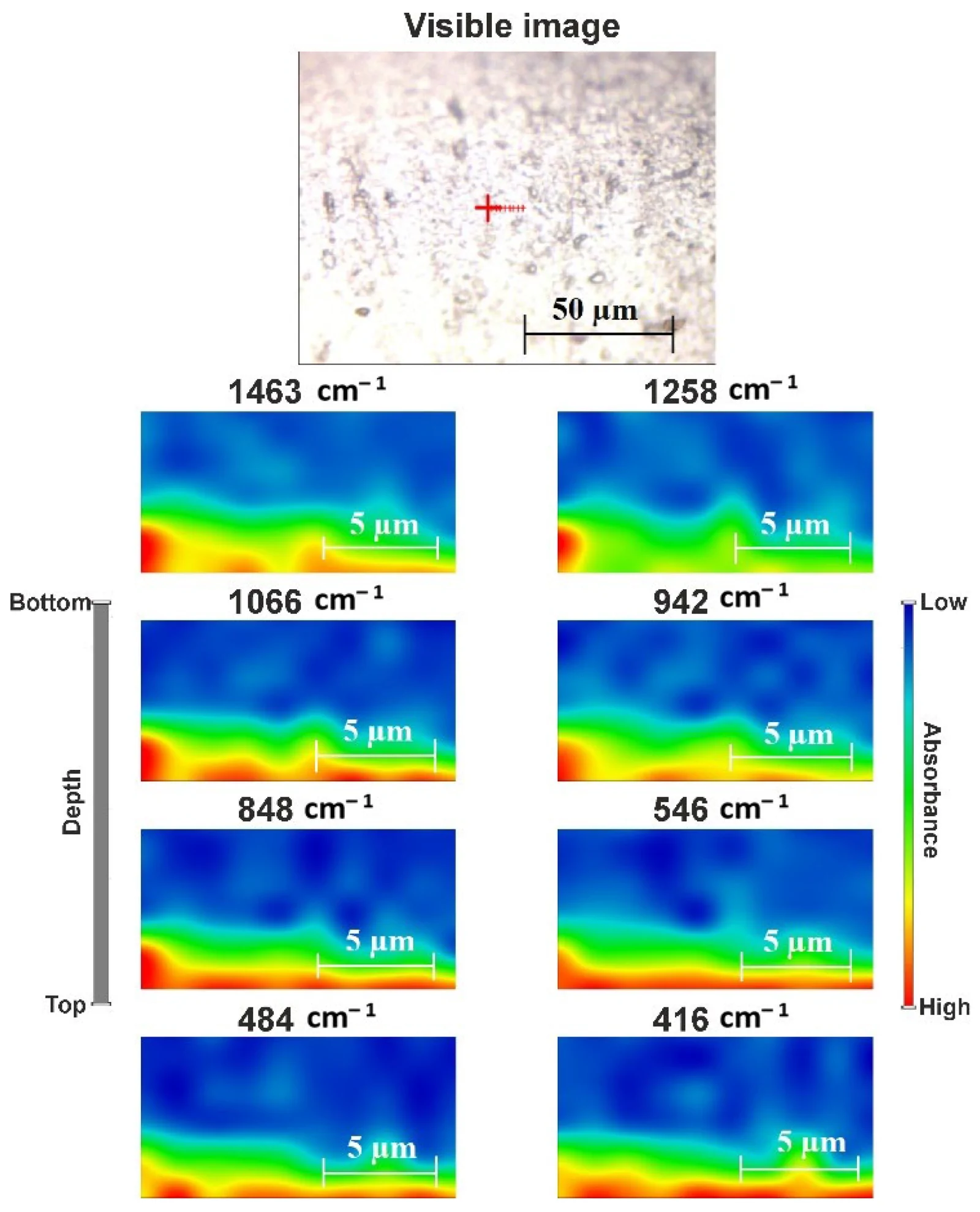
Confocal Raman microscopic visible image and Raman depth profiling of compound distribution in the α-1,3-glucan film. The red graduated line with a cross in the visible image indicates the mapping site.

The next step in the structural analysis was Raman spectroscopic mapping to examine the distribution of selected chemical groups in the film (Figure 5). The microspectroscopy revealed a uniform and regular spatial distribution of chemical compounds within the sample. A low content of mixed C-H, O-CH_3_, H-CH, HOC (1463 cm^−1^), C(=O)O (484 cm^−1^), and HCC (416 cm^−1^) groups was detected, while the highest intensity and concentration of the C-H group associated with α-glycosidic bonds (848 cm^−1^) were observed. To examine the confocal distribution of chemical groups within the film, depth profiling was performed (Figure 6). For all the studied chemical groups, intensity decreased with depth. Notably, differences in the chemical composition were observed between the surface and deeper layers of the sample, suggesting either denser packing near the surface or surface roughness (Figure 2). However, it is important to note that resolution and signal throughput also decrease with depth, which is a limitation of confocal microscopy and could potentially lead to misinterpretation of the results [45].

**Figure 7:**
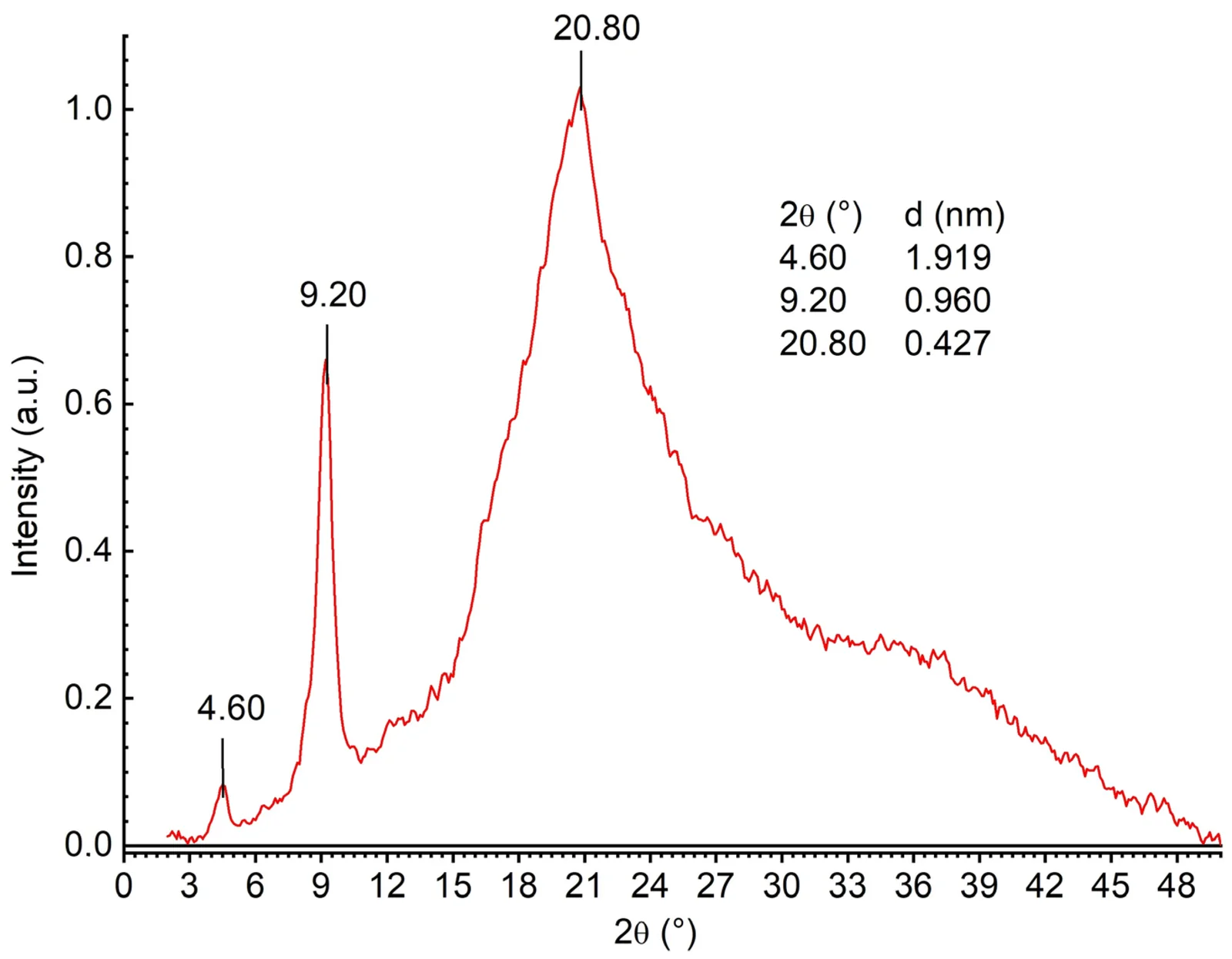
Wide-angle X-ray diffraction pattern of the α-1,3-glucan film with calculated interplanar spacing (d) values.

The wide-angle X-ray diffraction (WAXD) pattern of the α-1,3-GLU film exhibited three distinct peaks at 4.60°, 9.20°, and 20.80° (Figure 7), indicating a semi-crystalline structure. These peaks correspond to specific interlayer spacings (d), i.e., the distances between crystallographic planes within the film. The largest d (1.92 nm) likely represents the primary interlayer spacing, while the smaller values (0.96 nm and 0.43 nm, Figure 7) correspond to higher-order reflections or secondary planes. The degree of crystallinity (Xc) of the film was approximately 23.1% (Table 3), suggesting that the amorphous phase dominates in the α-1,3-GLU film. The relatively low amount of crystalline structure can be attributed to the presence of glycerol (plasticizer), disrupting the molecular organization and favoring an amorphous structure [46]. It should be mentioned, however, that the WAXD pattern of α-1,3-GLU (powder) also shows a broad peak in the range from 15° to 35°, indicating the presence of mixed amorphous and crystalline domains in its structure [47].

### 2.3. Physiochemical Properties of the Film

**Figure 8:**
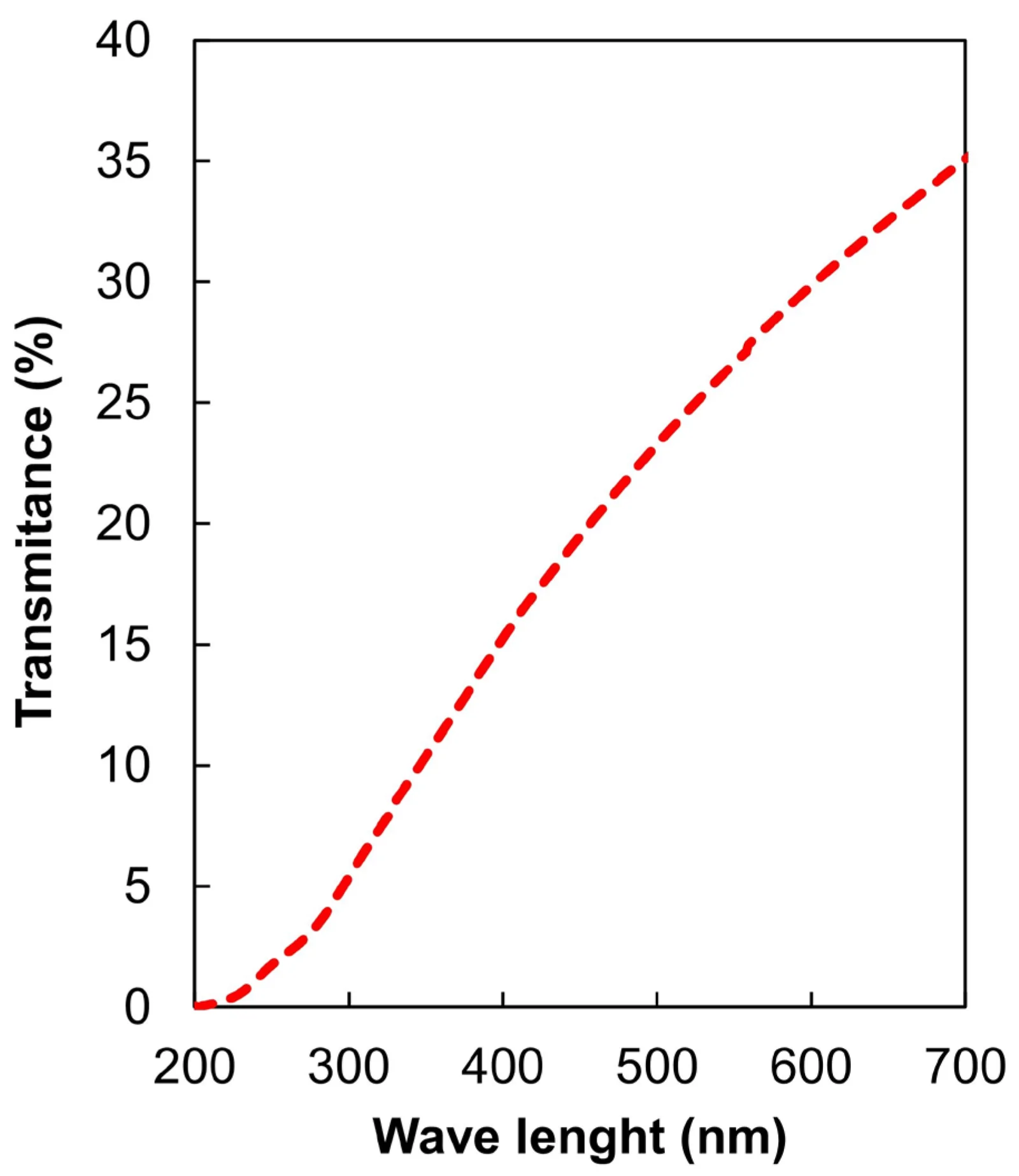
UV/VIS light transmission of the α-1,3-glucan film.

As can be seen in Figure 8, the light transmission (LT) of the α-1,3-GLU film linearly decreased with the decrease in the wave length. Consequently, the film provided a better barrier against UVC light (LT ≤ 3.4%) compared to UVB (LT_280–315 nm_ ≈ 3.4–15%) or UVA (LT_315–400 nm_ ≈ 7–15%). It is worth mentioning that, considering these values, the α-1,3-GLU film is a much better UV light absorber than films based on different types of starch, pullulan, chitosan, or cellulose derivatives [22,24,48,49]. With its low visible-light transmission (LT_400–700 nm_ = 15–35%), the α-1,3-GLU film exhibited high opacity (Op), as confirmed by both the macroscopic observation (Figure 1F) and the spectrophotometric measurements (Op = 5.65 Abs_600_/mm, Table 3). For comparison, the Op of many polysaccharide-based films is about 5-10 times lower [22,48,49]. Since large crystals in polymer materials scatter light and cause Op [50], the extensive crystal network of the coagulated polymer (Figure 1E) was likely responsible for the hazy appearance of the film (Figure 1F, Table 3). With regards to the L*a*b* parameters (Table 3), they indicated that the film had a white-yellowish color, which reflected the color of the FFS (Figure 1A,B).

Surprisingly, the α-1,3-GLU film was perfectly neutral (pH = 6.96, Table 3). This result may have been partly due to its high water content (46.27%, Table 3), reflecting significant absorption and retention of the glycerol solution used for the re-plasticization. The strong hygroscopic nature of glycerol, attributed to its three hydroxyl groups, enables it to act as a humectant [51], retaining water in the polymer matrix and/or absorbing moisture from the air [52]. Consequently, a higher glycerol content in the film matrix typically results in a higher moisture content (MC) [53]. In line with the present findings, a high MC (>40%) is a characteristic feature of over-plasticized films (e.g., those with a polymer–glycerol ratio ≥ 1:0.8) [54]. It is worth mentioning that, typically, the MC of biopolymeric films that are reasonably plasticized with glycerol ranges from 10 to 25% [22,24,52].

The water activity (a_w_ = 0.48, Table 3) suggested that the water in the α-1,3-GLU film was partially free. With a_w_ < 0.6, the film can could be considered as microbiologically stable, as bacteria, yeasts, and molds require higher water activity to proliferate. The lower film a_w_ compared to the 40% glycerol solution (~0.86 [55]) used for re-plasticization resulted likely from water removal during drying and glycerol binding within the polymer matrix, which reduced moisture interaction.

The α-GLU film demonstrated hydrophilic characteristics, as evidenced by its water contact angle (WCA) of 53.56° (Table 3), which was well below the 90° threshold typically used to distinguish hydrophilic from hydrophobic surfaces. The obtained value indicated that the α-GLU film had moderate wettability, which was fairly similar to that of some other biopolymer materials. For comparison, the WCA of glycerol-plasticized films made from potato starch, sodium caseinate, and polysaccharide–gelatin blends was 46.73–59.10°, 67.55°, and 38.51–45.46°, respectively [28,52]. The surface energy (γ_L_) of the film was calculated to be 116.03 mN/m (Table 3), which reflected its extremely strong (for a biopolymeric material) affinity for polar substances. For comparison, the γ_L_ of potato starch films plasticized with increasing concentrations of glycerol ranged from 46.10 to 59.10 mN/m, while common plastics typically exhibit γ_L_ values of ~20–40 mN/m [56,57]. The high surface energy of the α-1,3-GLU film could be explained by the significant number of hydroxyl groups both in the polysaccharide and in the plasticizer.

**Figure 9:**
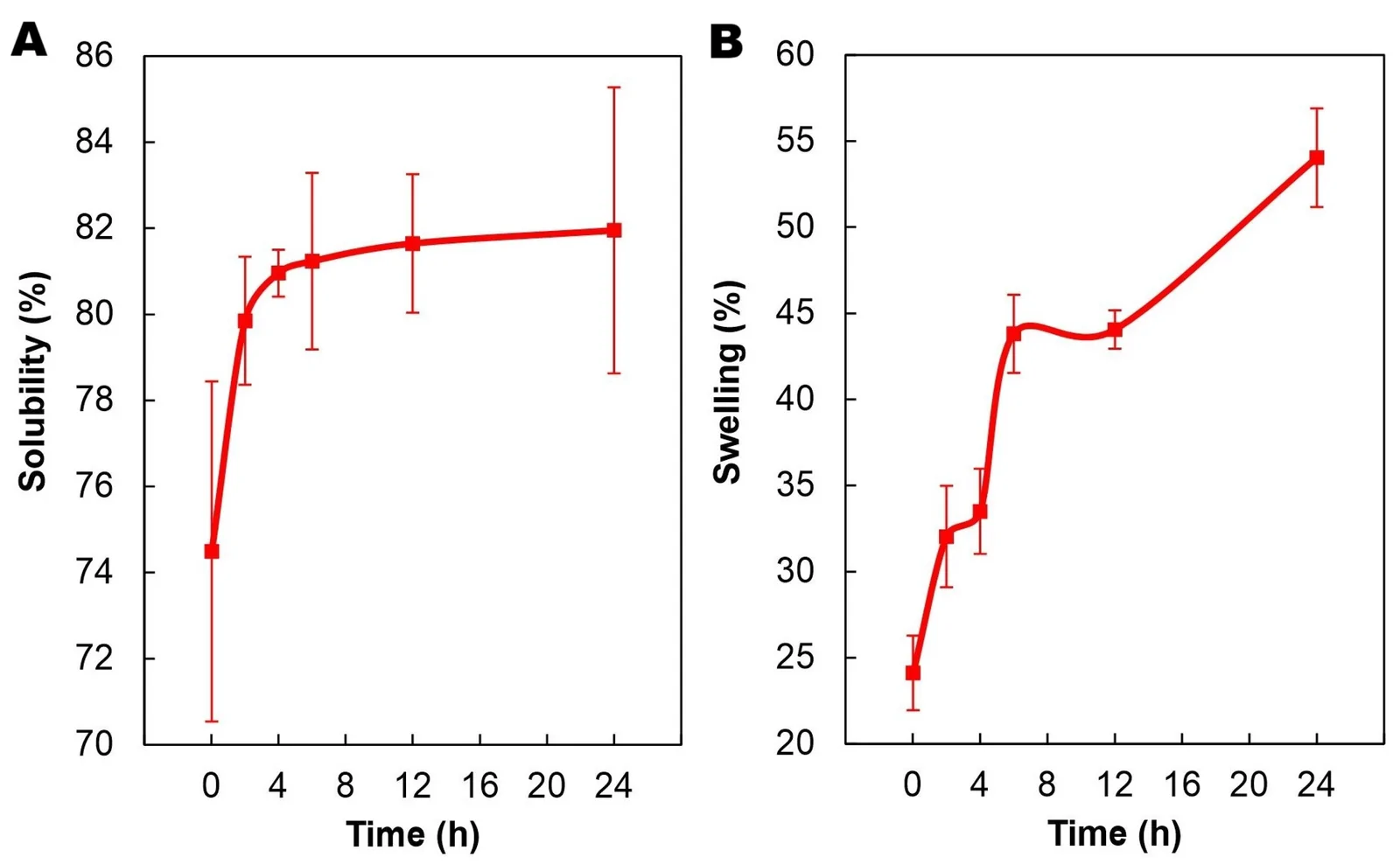
Solubility (**A**) and swelling (**B**) ratio of the α-1,3-glucan film.

Contrary to expectations, the α-1,3-GLU film was highly prone to dissolution upon contact with water (74.49% after 1 min, Figure 9A). Since α-1,3-GLU represents a water-insoluble fraction and half of the film’s mass consisted of water, this high solubility (So) could be primarily attributed to the leaching of glycerol from the polysaccharide matrix. Namely, after re-acclimatization, the film remnants, which no longer contained hydrophilic glycerol, were unable to retain or absorb moisture from the surrounding environment. As a result, their mass decreased significantly compared to before the test, leading to a high So. Fast dissolution was likely due to weak film compactness (Figure 1E), which facilitated rapid water penetration in water/glycerol-rich areas.

Over time, the film absorbed increasing amounts of water (Figure 9B), likely due to the gradual loosening of its structure and the entrapment of water in the micro-spaces within the network formed by α-1,3-GLU (Figure 1E). Due to the low water affinity of α-1,3-GLU, combined with a high MC and rapid So in water (Figure 9A), the film exhibited limited water uptake (24.11–54.03%, depending on the contact time, Figure 9B). Usually, biopolymer-based films absorb water in an amount of several times their initial weight; for example, swelling (Sw) of glycerol-plasticized starch film can reach ≈250%, while the Sw value of film made of pullulan is several thousand % (at least before it completely disintegrates in water) [24].

With a water vapor permeability (WVP) value of 53.69 ± 3.67 g mm m^−2^ d^−1^ kPa^−1^(Table 3), the α-1,3-GLU film does not stand out from many previously prepared and described glycerol-plasticized polysaccharide films. Specifically, when the polysaccharide concentration in the FFS is 5% and the “wet cup” method is employed to determine the barrier properties (as in this study), the film’s WVP typically falls within the range of 37–65 g mm m^−2^ d^−1^ kPa^−1^ [22,23,48,58]. Therefore, taking into account the standard deviation (Table 3), the WVP of the α-1,3-GLU film is quite similar to that observed for films based on carboxymethyl cellulose, methylcellulose, corn starch, pullulan, gum Arabic, or water-soluble soy polysaccharides [23,49]. This comparison, however, must be interpreted with caution, as the aforementioned polysaccharides were plasticized with varying levels of glycerol, which, as is known, dramatically changes the WVP of the resulting films [59].

The α-1,3-GLU film exhibited very poor mechanical strength, stretchability, and stiffness (Table 3), which disqualifies it for use in packaging applications requiring mechanical integrity of the material. These results are likely due to the soaked state of the film, i.e., its high MC (Table 3) and glycerol content. Additionally, the mechanical weakness of the film could be attributed to its non-coherent microstructure (Figure 1) and the dominant presence of the amorphous phase (Figure 7, Table 3). When comparing the data with previous studies, the tensile strength (TS) and elongation at break (EB) values of the α-1,3-GLU film (Table 3) are similar to those found for glycerol-over-plasticized whey protein and pea protein films [54,60]. However, it should be noted that the α-1,3-GLU film is not significantly mechanically weaker than some highly glycerol-plasticized starch films (polymer–glycerol ratio ≈ 1:0.5) with TS ≈ 2 MPa [52,61,62]. Nevertheless, the significant mechanical weakness of the α-1,3-GLU film becomes evident when its TS is compared to the respective values of materials based on pullulan (polymer–glycerol ratio = 1:0.2, TS ≈ 15 MPa) [58], methylcellulose (polymer–glycerol ratio = 1:0.4, TS ≈ 26 MPa) [23], carboxymethyl cellulose (polymer–glycerol ratio = 1:0.2, TS ≈ 42 MPa) [49], or insufficiently plasticized corn starch (polymer–glycerol ratio = 1:0.17, TS ≈ 40 MPa) [24].

### 2.4. Action of Microorganisms on the Film

**Figure 10:**
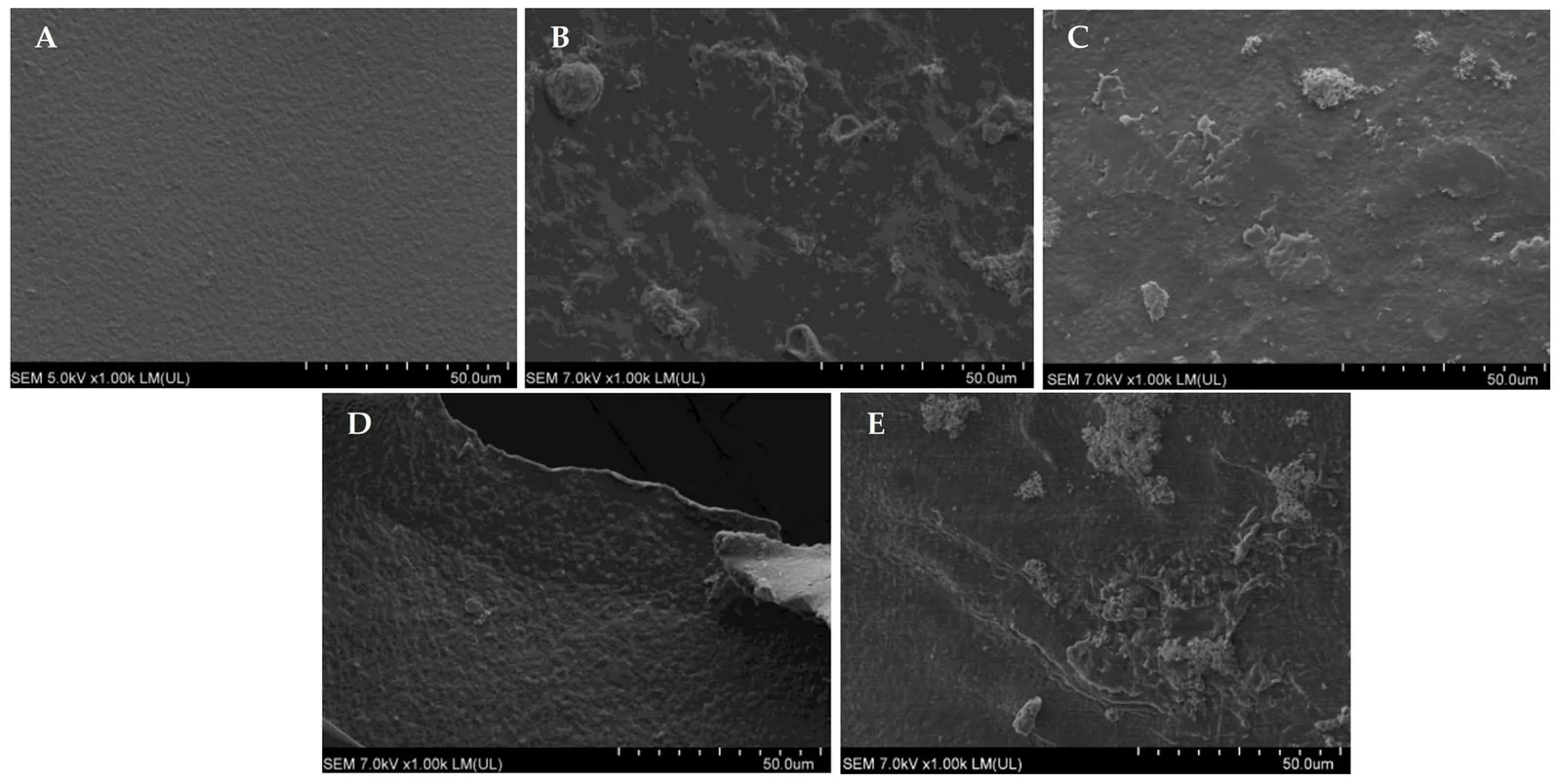
Scanning electron microscopy microtopographies (magnification ×1000) of the α-1,3-glucan film before incubation (**A**) and after incubation with a soil–water mixture (1:9) (**B**) loaded with *Bacillus subtilis* (**C**), *Escherichia coli* (**D**), and *Staphylococcus aureus* (**E**).

Scanning electron microscopy (SEM) (Figure 10A) confirmed the observations made using DIC microscopy (Figure 2), showing that, before testing, the film surface was slightly rough. After five days of incubation in a soil–water (1:9) mixture, the film was in a swollen state (consistent with previous observations, Figure 9B), while retaining its structure and showing no signs of degradation (no cracks or flaking surface). However, some round and convex structures of different sizes were visualized, suggesting the adhesion of microorganisms of soil origin (Figure 10B). In the case of the film incubated in the soil–water mixture loaded with *Bacillus subtilis*, a significant portion of the surface was covered with small round objects, likely the bacteria. Moreover, patch-like structures were visible, suggesting decomposition of the film (Figure 10C). A similar observation was made for the film incubated in the presence of *Staphylococcus aureus* (Figure 10E). More uniformly packed biofilm was observed in the film incubated in the soil–water mixture with *Escherichia coli* (Figure 10D). In summary, the results revealed that the α-1,3-GLU film provided a good surface for the adhesion of soil microorganisms and the target Gram-positive (*B. subtilis*, *S. aureus*) and Gram-negative (*E. coli*) bacteria. The likely cause was the high MC of the film (Table 3) (partly due to its swollen state (Figure 9B)) and its good wettability and hydrophilicity (high WCA and γ_L_, Table 3), which promoted bacterial adhesion. Additionally, the neutral pH of the film (Table 3), favored by the target bacteria, may have further supported their growth and contributed to material biodegradation. The percentage of the film surface colonized by these groups of bacteria was approximately 25% and 50%, respectively. The adhesion of these microorganisms to the film could indicate their ability to break it down by enzymes, similar to the biodegradation processes seen with many naturally occurring polymers [63,64].

### 2.5. Susceptibility of the Film to Degradation

The *Trichoderma* genus consists of avirulent filamentous fungi commonly found in soil, as well as in root and foliar ecosystems. In this study, *Trichoderma harzianum* was selected as a model organism for the biodegradation analysis of the α-GLU-based film due to its proven ability to produce α-1,3-glucanase [65]. It is also worth mentioning that *T. harzianum* synthesizes a range of hydrolytic enzymes capable of breaking down not only polysaccharides [66] but also diesel fuel [67] and some plastics [68].

**Figure 11:**
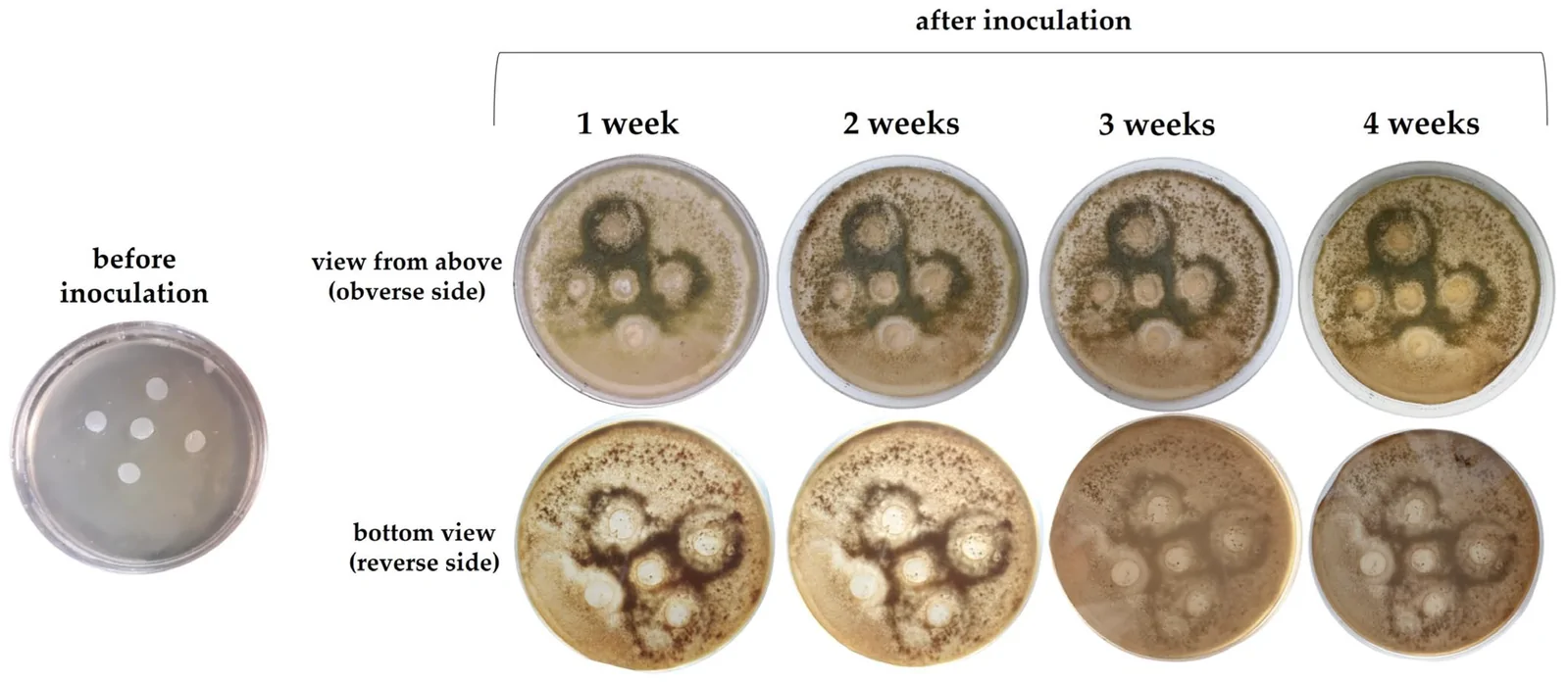
Appearance of PDA medium inoculated with *Trichoderma harzianum* in the variant with the α-glucan film disks placed on the surface of the medium.

**Figure 12:**
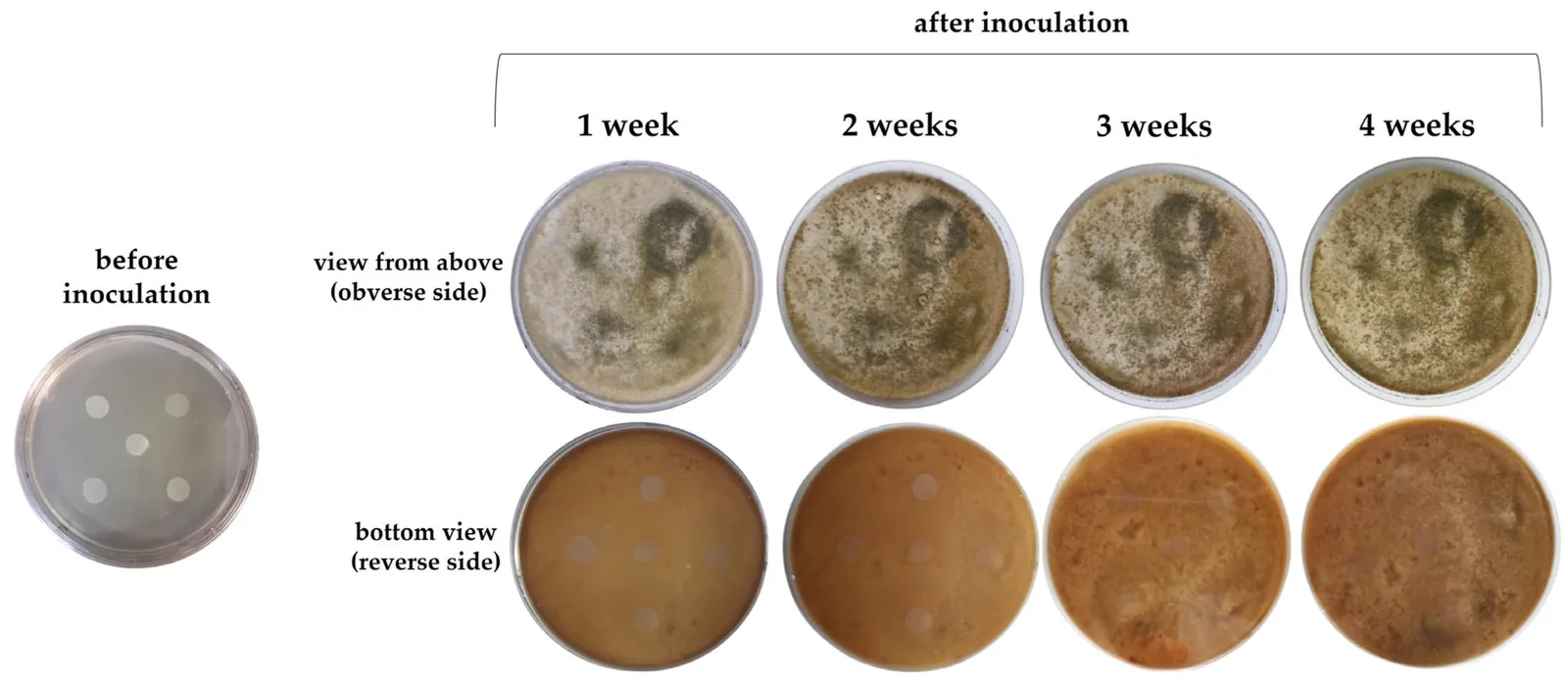
Appearance of PDA medium inoculated with *Trichoderma harzianum* in the variant with the α-glucan film disks submerged in the medium.

After just one week of the experiment, both the younger, developing mycelium (gray) of *T. harzianum* and the later growth stage producing conidia (green) were observed in both the medium where the films were placed on the surface (Figure 11) and the medium in which they were submerged (Figure 12). The presence of circular zones of green mycelium surrounding the film samples, rather than directly adhering to them (Figure 11), suggested that certain film components, e.g., glycerol, diffused into the medium, creating a zone of influence. As demonstrated, glycerol serves as an important carbon source that supports the growth and survival of *T. harzianum* [69,70] and the production of cellulases [71]. Unfortunately, the effect of glycerol on α-1,3-glucanase synthesis remains unclear and requires further investigation. The surface of the medium containing the submerged films also showed green mycelium nearby (Figure 12). Overall, the fungus more intensively colonized the medium in this variant of the experiment: i.e., the mycelium was visible on the surface and eventually on the underside of the plates. This could have been due to the strong contact between the film disks and the solidifying medium, which likely promoted the diffusion of the film components, including glycerol. As presented in Figure 9A, the film exhibited a high tendency to dissolve.

As widely recognized, *Trichoderma* species are aerobic organisms that flourish primarily on the surface layers of the substrate [72]. Therefore, the air-exposed side was more intensively colonized than the bottom part of the medium (Figure 11 and Figure 12). The limited availability of oxygen may explain the lack of mycelial growth beneath the films (in the “surface” variant, Figure 11). Although the intense growth of the mycelium made macroscopic observation of the film samples difficult after 2 and 4 weeks of incubation (Figure 12), a clear reduction in sample size was observed by the end of the experiment in the submerged films, which may have indicated the hydrolysis of α-1,3-GLU by enzymes produced by *T. harzianum*. To determine this, further studies with the enzymes were undertaken to gain a better understanding of the susceptibility of the α-1,3-GLU film to degradation. The results in Table 4 demonstrate that both bacterial endo-α-1,3-glucanase and fungal (*T. harzianum*-origin) exo-α-1,3-glucanase effectively hydrolyzed the α-1,3-GLU film. While their activity was comparable in the initial stages of hydrolysis, after 24 h, exo-α-1,3-glucanase exhibited significantly greater efficiency, leading to 17.16% degradation, whereas endo-α-1,3-glucanase caused only 11.12% breakdown (Table 4).

The observed degradation efficiency was likely partially attributable to the film’s amorphous structure, as the lack of crystalline order facilitated microbial and enzymatic access to susceptible chemical bonds. This structural characteristic, combined with the film’s high So (Figure 9A), promoted both nutrient diffusion and enzymatic hydrolysis, ultimately contributing to the breakdown of the α-1,3-GLU film.

## 3. Materials and Methods

### 3.1. Preparation of α-1,3-GLU

α-1,3-GLU was isolated from the fruiting bodies of *Laetiporus sulphureus* according to a previously described procedure [73]. The fungal specimens were collected from infected trees in the vicinity of Lublin (Lublin, Poland, 51°15′00″ N, 22°34′00″ E). NaOH pure p.a. was obtained from POCH S.A. (Gliwice, Poland), and glycerol (≥99.0%) was purchased from Sigma-Aldrich (Burlington, MA, USA).

### 3.2. Optimization of Plasticizer Concentration and Heating Temperature

The 1M NaOH solutions containing α-1,3-GLU (5% *w*/*w*) and increasing concentrations of plasticizer (glycerol at 1, 2, 3, 4, and 5% *w*/*w*) were tested to determine their ability to produce non-cracked, peelable, and flexible films. Briefly, all components were mixed at room temperature for 1 h using a Multi RS-60 rotator (Biosan, Riga, Latvia). The film-forming solutions (FFSs) were then degassed using a stainless steel tea strainer mesh sieve, cast onto Teflon-coated trays in an amount of 0.139 g/cm^2^, and dried at ~25 °C and ~50% relative humidity (RH) for 24 h. The optimal plasticizer level (4%) was determined based on sensory observations of the resulting materials. For analytical purposes, the FFSs containing 4% (*w*/*w*) glycerol were also heated at higher temperatures (30–90 °C) for 20 min.

### 3.3. Film Preparation

The film was prepared from an alkaline solution containing α-1,3-GLU (5% *w*/*w*) and glycerol (4% *w*/*w*), as described in Section 3.2, using Teflon-coated trays with an area of either 144 cm^2^ or 4 cm^2^, depending on the test. Subsequently, to leach out NaOH, the film samples were soaked in water (7 mL/cm^2^, 20 °C, 15 min) with gentle mixing (50 rpm) using an ES-60 incubator (MIULAB, Hangzhou, China). Rinsing was performed twice: i.e., until the pH of the water remained unchanged. The samples were then drained and re-plasticized by soaking in a 40% (*w*/*w*) aqueous glycerol solution for 2 h at 20 °C with gentle mixing (50 rpm). The excess solution was removed by gently blotting the film surface with filter paper until an equilibrium was reached. The 40% glycerol concentration in the bath, i.e., the minimal level enabling the production of testable (non-rigid and non-sticky) material, was determined in a preliminary study.

### 3.4. Analysis of Film Properties

#### 3.4.1. Film Thickness and Conditioning

The obtained films were cut into samples, and their thickness was measured using a 547–401 digital thickness gauge (Mitutoyo, Tokyo, Japan). Prior to testing, the samples were stored in an MLR-350 climatic test chamber (Sanyo Electric Biomedical Co., Ltd., Oizumi-Machi, Japan) for 48 h at 25 °C and 50% RH.

#### 3.4.2. Microstructure

The morphology of the FFS and the film was observed using a CKX53 microscope (Olympus, Tokyo, Japan) and a LEICA 5500B microscope (Leica Microsystems GmbH, Wetzlar, Germany) with a differential interference contrast (DIC) optical system. The 2D DIC images were converted into 3D topographies using ImageJ 1.54j software to assess the air-side surface roughness of the film, measured as root-mean-square roughness (Rq) [32].

#### 3.4.3. Attenuated Total Reflection–Fourier Transform Infrared (ATR-FTIR) Spectroscopy

The FTIR spectrum of the film was recorded in the region of 4000–750 cm^−1^ using a Nicolet 6700 FT-IR spectrometer (Thermo Scientific, Waltham, MA, USA) with the ATR mode. Three film samples were scanned, and the spectra were averaged. Each spectrum represented 120 scans with a resolution of 4 cm^−1^. The obtained spectra were normalized, baseline-corrected, and analyzed using GRAMS/AI software (ThermoGalactic Industries, Keene, NH, USA). A final spectrum was obtained using OMNIC 8.2.0.387 software (Thermo Fisher Scientific, Madison, WI, USA).

#### 3.4.4. Raman Spectroscopy and Surface Mapping

The Raman spectra were obtained in the range of 150–2000 cm^−1^ using a DXR confocal Raman microscope and a X–Y-Z motorized sample stage (Thermo Electron Scientific Instruments LLC, Madison, WI, USA). The microscope was coupled with a charge-coupled device camera (Omron Sentech Co., Ltd., Ebina, Japan) with a 0.8 megapixel image sensor. The excitation laser wavelength was 780 nm. The mapping measurements were carried out using a long working distance ×50 objective without photobleaching. All spectra and maps–spatial distribution and depth profiles (sampling point spread along X, Y, and Z axes) were obtained with an exposure time of 10 s with laser power set to 15 mW, and 10 exposures per point using an operating spectral resolution of 4 cm^−1^ of Raman shift. A 50 μm pinhole aperture was used. The autofocus at each map point was used in the case of height-diverted samples. X-Y mapping consisted of 10 × 10 single measurement points with a step size of 2 µm. In the confocal maps, the step between the measurement points was 1.5 μm in both X and Z directions. The linear depth profiles were set to 10 × 10 points in both the X and Z axes (in depth). The Z offset was 1.5 μm. All data processing and image assembly were performed using OMNIC 8.2.0.387 (Thermo Fisher Scientific, Madison, WI, USA) and CytoSpec ver. 2.00.01 (CytoSpec, Berlin, Germany) software. Five spectra of film samples were recorded, baseline-corrected, and averaged before analysis. The final spectrum was smoothed with the Savitzky–Golay algorithm (13 smoothing points) in GRAMS/AI software (ThermoGalactic Industries, Keene, NH, USA).

#### 3.4.5. Wide-Angle X-Ray Diffraction (WAXD)

The X-ray diffraction pattern for the film was recorded using a URD 6 Seifert X-ray diffractometer (FPM-Seifert, Freiberg, Germany) with a CuKα radiation source. The measurement settings included a current of 30 mA, a voltage of 40 kV, and a diffraction angle (2θ) range from 2° to 50°, with a step size of 0.1° and a scanning rate of 0.1° per 15 s at approximately 25 °C. Triplicate scans were performed for each film to ensure high reproducibility.

Using Bragg’s law (1), the interlayer spacing (d, nm) was calculated for each peak individually.

(1) $$d=\frac{\lambda }{2\sin\theta }$$

where λ is the wavelength of the CuKα X-ray radiation (0.154 nm), and θ is the angle of incidence (°), i.e., half the value of 2θ [74].

The obtained diffraction pattern was deconvoluted using Gaussian–Lorentzian peak fitting to identify the crystalline content and the amorphous background. Data analysis was performed using OriginPro 2018 (OriginLab, Northampton, MA, USA). The degree of crystallinity (Xc, %) was determined using Equation (2):

(2) $$X_{c}=\frac{A_{c}}{At}\times 100$$

where *Ac* is the area under crystalline peaks, and *At* is the total area under the diffraction pattern, including both crystalline and amorphous contributions [75].

#### 3.4.6. Physicochemical Properties

The pH, optical parameters (L*a*b* values, light transmission (LT, %), and opacity (Op, Abs_600_/mm)), moisture content (MC, %), and mechanical properties (tensile strength (TS, MPa), elongation at break (EB, %), elastic modulus (EM, MPa), and puncture strength (PS, MPa)) of the film were determined as described previously [49,76].

The water activity (aw) of the film (≈1 g) cut into pieces was determined using the LabTouch-aw meter (Novasina, Lachen, Switzerland). The water contact angle (WCA) was determined as described previously [77]. The surface energy (γ_L_, mN/m) of the film was calculated using the Young–Dupré Equation (3):

(3) $$\gamma_{S}=\gamma_{L}\left(1+cosCA\right)$$

where *γ_L_* is the surface tension of water (72.8 mN/m), and CA is the contact angle [78].

To assess the swelling (Sw, %) and solubility (So, %) rates, the film samples were shaken at 60 rpm using a Unimax 1010 shaker (Heidolph, Schwabach, Germany) in 30 mL of water at 25 °C for a duration between 1 min and 24 h [23]. The rate of water vapor transmission of the film was measured gravimetrically. Test tubes (with a flat rim and internal diameter = 14 mm) were filled with 10 mL of distilled water and tightly sealed with film disks (4 cm^2^) using hot-melt adhesive. The test tubes were placed in the test chamber set at 25 °C and 50% RH. The weight loss of the test tubes was monitored over 10 days, with weights recorded at 24 h intervals. The water vapor permeability (WVP) was calculated as described previously [49]. The mechanical properties were tested over 10 repetitions, while the other analyses were performed in three-to-five repetitions.

#### 3.4.7. Evaluation of the Action of Microorganisms

For determining the deterioration of the film due to the action of bacteria and soil microorganisms, the method described in ISO 846:2019 [79] was used. Horticultural soil was sieved through a 2 mm mesh sieve. Then, the concentration of microorganisms was determined with the serial dilution method (in the range of 10^−1^ − 10^−6^) using plate count agar medium (Oxoid Ltd., Basingstoke, UK). Briefly, the plates were incubated at 26 ± 1 °C for 5 days, and the final number of viable microorganisms was 10⁸ CFU/mL. Then, 10 g of the soil was mixed with 90 mL of sterile distilled water. The film samples (15 diameter disks) were sterilized for 15 min under UV light using an NV208B sterilizer (Beauty System, Wrocław, Poland), placed on sterile Petri dishes with a diameter of 90 mm, and flooded with 15 mL of a soil–water mixture.

Moreover, soil–water mixtures loaded with *Escherichia coli* ATCC 8739, *Staphylococcus aureus* ATCC 6538P, and *Bacillus subtilis* ATCC 6633 were used for incubation. Briefly, the bacteria were cultured for 48 h at 29 °C (*B. subtilis*) and 35 °C (*E. coli* and *S. aureus*) on tryptone soya agar (Oxoid Ltd., Basingstoke, UK). A suspension of the strains in saline was prepared to a turbidity equivalent to 0.2 on the McFarland scale. Then, 1 mL of the suspension was separately added to 10 mL of the soil–water mixture.

The Petri plates were incubated for 5 days at 29 °C (*B. subtilis*) and 35 °C (*E. coli* and *S. aureus*) with an RH of 95% ± 5% in a climatic chamber. After exposure, the film samples were cleaned according to ISO [79] and then analyzed using a scanning electron microscope SU 8010 (Hitachi, Tokyo, Japan). Before observation, the samples were sputtered with a 1 nm gold layer using a 108 auto-sputter coater equipped with an MTM-10 thickness monitor (Cressington Scientific Instruments, Watford, UK). Images were captured at a magnification of ×1000. A grid was used to evaluate the percentage of colonization of the film surface by soil microorganisms and the target bacteria, following ISO [79].

#### 3.4.8. Susceptibility of the Film to Degradation by Trichoderma harzianum

Two approaches were used to assess the susceptibility of the film to microbial attack by *Trichoderma harzianum* CBS 436.95 (mushroom compost fungus): (i) 10 mm diameter film disks (≈12 mg) were placed on the surface of solidified potato dextrose agar (PDA) medium, and (ii) liquid PDA medium was poured over the film disks in Petri dishes immediately after autoclaving. A Petri dish with the PDA medium but without film disks served as a control. The plates were inoculated with a suspension of *T. harzianum* conidia at a concentration of approximately 2 × 10^5^ conidia/mL in phosphate-buffered saline and incubated at room temperature (≈22 °C) for four weeks. Susceptibility to fungal attack was assessed through visual observations of the film samples and mycelium growth on both the upper and lower sides of the medium. Both experimental variants were performed in triplicate using five film samples per Petri dish.

#### 3.4.9. Enzymatic Breakdown of the Film

Two α-1,3-glucanases were used to analyze the susceptibility of the film to enzymatic hydrolysis: (i) a bacterial α-1,3-glucanase (EC 3.2.1.59) produced by *Paenibacillus curdlanolyticus* MP-1, with endolytic activity, and (ii) a fungal α-1,3-glucanase (EC 3.2.1.84) produced by *Trichoderma harzianum* CBS 436.95, demonstrating exolytic activity. The endo-α-1,3-glucanase was obtained following the protocol described by Pleszczyńska et al. [80], while the exo-α-1,3-glucanase was prepared using the method described by Wiater et al. [81]. The endo-α-1,3-glucanase was suspended in 50 mM potassium phosphate buffer (pH 6.5) and the exo-α-1,3-glucanase in 0.2 M acetate buffer (pH 5.5), both at 0.1 U/mL. The film diskswere placed in the wells of a 6-well polystyrene plate (Nest Biotechnology Co., Ltd., Wuxi, China) and then covered with 5 mL of the α-1,3-glucanase solutions or the buffers (controls) and incubated at 40 °C for 24 h with gentle shaking (using a rocking shaker). After 1, 2, 3, 6, and 24 h, the released reducing sugars were quantified using the Somogyi–Nelson method [82,83] with d-glucose as a standard. The hydrolysis experiments were performed in triplicate. Faster growth of reducing sugars in the reaction mixture indicated higher susceptibility to enzymatic degradation.

## 4. Conclusions

In this study, a method for producing film from a water-insoluble/alkali-soluble polysaccharide, α-1,3-GLU from *L. sulphureus*, was proposed. Since it was observed that α-1,3-GLU does not completely dissolve in 1M NaOH at a concentration of 5%, other methods to improve its solubility should be explored. Heating, however, should be avoided, as it leads to undesirable browning. Since the rinsing step removed not only NaOH but also the plasticizer (glycerol), re-plasticization was necessary. Unfortunately, this process yielded a moist film (water made up about half of its mass) with very poor mechanical properties, disqualifying it from packaging applications which require material integrity. Unlike α-1,3-GLU, the film was highly soluble in water, partially likely due to the flushing out of the glycerol and loss of moisture absorbed during re-plasticization. Despite the relatively high water content (as for a biopolymer material), the film had an a_w_ value of less than 0.6, which suggests that it might be potentially resistant to microbial growth without the need for additional preservatives. The high surface energy suggests that this material can interact well with polar substances, making it suitable for biomedical applications, coatings, or adhesive layers. The biodegradability of the film should not be a concern. Both the soil microorganisms and the tested bacteria were capable of growing on the α-1,3-GLU film. *T. harzianum* was able to utilize the film as a carbon source—initially likely due to the presence of glycerol and later by consuming α-1,3-GLU itself. Additionally, the film was found to be more susceptible to hydrolysis by exo-α-1,3-glucanase than by endo-α-1,3-glucanase. The observed degradation efficiency was likely due to the film’s amorphous structure, which, along with its high solubility, wettability, hydrophilicity, and neutral pH, facilitated microbial biofilm formation and enzymatic access to susceptible chemical bonds, promoting nutrient diffusion and enzymatic hydrolysis.

Research on α-1,3-GLU films should be continued to further optimize their composition, production methods, and functional properties and explore their potential. Especially, another method of plasticization is recommended, such as using substances which will not be removed during the NaOH washing process—for example, lipids. Improvements in transparency and mechanical properties, potentially through blending with other polymers, are also worth investigating. Such studies are crucial for advancing eco-friendly materials with applications in food packaging, medical coatings, and beyond, contributing to a reduction in the environmental impact.

## Figures and Tables

**Table 1:** The most prominent bands in the ATR-FTIR spectra of the α-1,3-glucan film and their corresponding assignments.

Wavenumber (cm^−1^)	Assignment *
3283	ν −OH
2935	ν_as_ -CH_3_, -CH_2_
2884	ν_s_ C-H
1651	ν C=O
1451	δ_as_ -CH_2_
1412	δ_s_ -CH_2_, -CH_3_
1345	ν C-H,C–C, -CH_3_
1243	τ_in-plane_ C–OH, C-H
1208	ν C–O, C–O–C (pyranose ring)
1145	ν_as_ C–O–C, C-C (glycosidic linkage)
1106	ν C-O
1067	νC–O, CH_2_OH, τC-O
1029	ν C-O (α-glycosidic bond)
926	δ C–H (α anomer)
850	τ_out-of-plane_C-H of *α*-glucan units

* ν—stretching vibrational mode, δ—deformational, τ—bending, s—symmetrical, and as—asymmetrical modes.

**Table 2:** Assignment of the α-1,3-glucan film’s Raman bands.

Raman Shift (cm^−1^)	Assignment *
1519	τ_in-plane_ -CH_2_
1463	τ C-H, O-CH3, H-CH, HOC
1392	τ C-H
1339	ν_s_ COO−
1258	δ C-CH
1221	ν C-H (rings)
1112	ν C-O-C
1086	ν C-C, C-O
1066	ν_s_ C-O
973	ν C-C, C-O-C
942	ν C-H (rings)
924	δ _out-of-plane_ C-H
848	τ C-H (α-glycosidic linkage)
818	τ _out-of-plane_ C-H
717	τ _out-of-plane_ C-H, νs C-H
671	τ _out-of-plane_ C-H
576	δ C-C-C
546	δ _out-of-plane_ C=O
484	τ C(=O)O
435	τ _in-plane_ C-C=O
416	τ _out-of-plane_ HCC
380	δ C-C(=O)C
327	τ _out-of-plane_ C-C-C-C
284	δ C-C-C
258	δ -OH
212	δ _in-plane_ C-C-C
176	δ C-C

* ν—stretching vibrational mode, δ—deformational, τ—bending, and s—symmetrical mode.

**Table 3:** Thickness, root-mean-square roughness (Rq), crystallinity (Xc), pH, opacity (Op), L*a*b* values, moisture content (MC), water activity (a_w_), water contact angle (WCA), surface energy (γ_L_), water vapor permeability (WVP), tensile strength (TS), elongation at break (EB), elastic modulus (EM), and puncture strength (PS) of the α-1,3-glucan film.

Properties	Values
Thickness	98.38 ± 7.69
Rq	26.24 ± 3.12
Xc (%)	23.1± 1.67
pH	6.96 ± 0.06
Op (Abs_600_/mm)	5.65 ± 0.72
L*	90.47 ± 0.80
a*	−0.45 ± 0.12
b*	−5.01 ± 0.23
MC (%)	46.86 ± 2.40
a_w_	0.48 ± 0.01
WCA (^o^)	53.56 ± 1.59
γ_L_ (mN/m)	116.03 ± 1.63
WVP (g mm m^−2^ d^−1^ kPa^−1^)	53.69 ± 3.67
TS (MPa)	1.28 ± 0.31
E (%)	10.09 ± 0.17
EM (MPa)	30.80 ± 3.18
PS	0.20 ± 0.03

**Table 4:** Effect of the hydrolysis time with α-1,3-glucanases on the amount of reducing sugars released from the α-1,3-glucan film and the percentage of film degradation.

Enzyme	Parameter	Hydrolysis Time				
		1 h	2 h	3 h	6 h	24 h
endo-α-1,3-glucanase	Released reducing sugars (mg)	0.065 ± 0.008	0.150 ± 0.011	0.359 ± 0.009	0.716 ± 0.012	1.368 ± 0.029
	Degradation (%)	0.53 ± 0.063	1.22 ± 0.092	2.92 ± 0.074	5.82 ± 0.099	11.12 ± 0.239
exo-α-1,3-glucanase	Released reducing sugars (mg)	0.106 ± 0.003	0.172 ± 0.013	0.269 ± 0.016	0.502 ± 0.016	2.111 ± 0.030
	Degradation (%)	0.86 ± 0.021	1.40 ± 0.105	2.19 ± 0.130	4.08 ± 0.133	17.16 ± 0.241

## Data Availability

The raw data supporting the conclusions of this article will be made available by the authors upon request.

## Abbreviations

The following abbreviations are used in this manuscript:

α-1,3-GLU	α-1,3-glucan
ATR-FTIR	attenuated total reflection–Fourier transform infrared
a_w_	water activity
CFUs	colony-forming units
DIC	differential interference contrast
EB	elongation at break
EM	elastic modulus
FFS	film-forming solution
LT	light transmission
MC	moisture content
Op	opacity
PDA	potato dextrose agar
PS	puncture strength
Rq	root-mean-square roughness
SEM	scanning electron microscopy
So	solubility
Sw	swelling
TS	tensile strength
WAXD	wide-angle X-ray diffraction
WCA	water contact angle
WVP	water vapor permeability
Xc	degree of crystallinity
γ_L_	surface energy
